# Hyperaldosteronism and Renal Artery Stenosis in a Post-Abdominal Aortic Aneurysm Patient: A Case Report

**DOI:** 10.5811/cpcem2022.6.56522

**Published:** 2022-08-08

**Authors:** Konnor Davis, Christopher J. Gilani, Gabriel Sudario

**Affiliations:** * University of California, Irvine, School of Medicine, Irvine, California; † University of California, Irvine, Department of Emergency Medicine, Orange, California

**Keywords:** abdominal aorta aneurysm, AAA, hyperaldosteronism, stent, case report

## Abstract

**Introduction:**

Patients with history of abdominal aortic aneurysm (AAA) undergoing surgical repair can have a myriad of surgical complications including compromise to large arteries branching from the aorta. Secondary hyperaldosteronism, characterized by high levels of aldosterone and renin, can be due to a multitude of causes, including renal artery stenosis, and presents with nonspecific symptoms of fatigue, increased thirst, and muscle spasms. While it can initially be difficult to diagnose given its multitude of metabolic abnormalities, secondary hyperaldosteronism is important to consider in patients presenting with uncontrolled hypertension, hypokalemia, and metabolic alkalosis.

**Case Report:**

This report explores the case of a 65-year-old male with a complicated medical history presenting to the emergency department with hypokalemia and hypertension six months after undergoing endovascular repair for an AAA and was found to have metabolic abnormalities including hypokalemia and metabolic alkalosis consistent with secondary hyperaldosteronism, likely secondary to renal artery stent stenosis. He was admitted to the hospital for four days and made a full recovery.

**Conclusion:**

This case highlights the need to understand, identify, and accurately diagnose hyperaldosteronism and recognize post-AAA repair complications of renal artery stenosis as a cause of this metabolic derangement.

## INTRODUCTION

An abdominal aortic aneurysm (AAA) is a weakening and balloon-like dilation of the abdominal aorta. Abdominal aortic aneurysms are often asymptomatic and undiagnosed until complications occur; ultrasound and computed tomography angiography (CTA) are essential to diagnosis. There are many risk factors for AAA including old age, being male, familial history, hypertension, dyslipidemia, and most importantly, smoking.^1^ In regard to the most serious AAA complication, a rupture accounts for roughly 150,000–200,000 deaths each year.^2^ Treatment of an AAA, which is not always indicated, includes open surgical repair (OSR) with placement of a straight or bifurcated graft or endovascular repair (EVAR) entering various arteries near the abdominal aorta to place covered stents.^1^,^3^ Each method has its own pros and cons; however, treatment is necessary because once an AAA ruptures, more than half of these patients will die before reaching the operating table and of those who do make it to surgery, mortality rates remain around 50%.^3^

Aldosterone is the main mineralocorticoid produced in the zona glomerulosa of the adrenal cortex. Aldosterone’s main function is to control electrolyte homeostasis and extracellular volume in the kidneys through sodium reabsorption and potassium and hydrogen ion excretion via urine.^4^ Aldosterone secretion is controlled by a myriad of factors including, but not limited to, angiotensin II, potassium, and adrenocorticotropic hormone.^4^ However, the principal regulator of aldosterone is the renin-angiotensin-aldosterone system (RAAS), which becomes activated in states of hypovolemia and renal hypoperfusion.^4^ Subsequently, aldosterone can be over- or under-secreted (hyper- and hypoaldosteronism, respectively) given various clinical conditions.

In this report we document a case of a previously unheard-of combination of complications of secondary hyperaldosteronism secondary to renal artery thrombosis as well as a focally stenosed renal artery stent following EVAR treatment for AAA. Despite the novelty of this presentation, the sequela of difficult to diagnose complications of hyperaldosteronism, especially in the presence of a patent stent following AAA repair, warrants discussion.

## CASE REPORT

A 65-year-old male with past medical history of hypertension, AAA without rupture status post endovascular repair (six months prior), mixed hyperlipidemia, and tobacco usage presented to the emergency department (ED) from an outside physician for an abnormal potassium of 2.3 millimoles per liter (mmol/L) (reference range: 3.5–5.1 mmol/L) and hypertension. On evaluation, he reported 2–3 months of polyuria and polydipsia that had progressively worsened. He reported needing to urinate hourly and was unable to sleep due to this symptom. He also reported generalized weakness and some constipation. He denied dysuria, abdominal or chest pain, shortness of breath, fever, cough, or diarrhea. Presentation vital signs showed a blood pressure of 195/122 millimeters mercury, oxygen saturation of 97% on room air, heart rate of 89 beats per minute, and respiratory rate of 18 breaths per minute.

Of note, the patient had been admitted six months prior with a non-ruptured AAA. He underwent aneurysm repair with aortoiliac stent graft and left renal artery snorkel placement/stenting. On exam, the patient was alert and oriented with no signs of trauma. Heart, lungs, and abdominal exam were all normal. Initial labs in the ED were notable for sodium of 133 mmol/L (reference range: 136–145 mmol/L), potassium of 2.4 mmol/L (3.5–5.1 mmol/L), chloride of 89 mmol/L (98–107 mmol/L), and hemoglobin of 17.6 grams per deciliter (g/dL) (12.0–16.0 g/dL). Venous blood gas revealed a pH of 7.53 (7.35–7.45), arterial bicarbonate of 38.5 mmol/L (21–27 mmol/L), and a total carbon dioxide of 40 mmol/L (36–42 mmol/L). Glomerular filtration rate was 55 mL/min/1.73 m^2^ and glucose was 129 milligrams per deciliter (mg/dL) (35–125 mg/dL). Urinalysis resulted in a urine protein of 9,087 (50–80 mg/day). On imaging, a CTA abdomen and pelvis showed chronic occlusion/thrombosis of the right main renal artery, moderate right renal atrophy, postsurgical changes of the endovascular repair, and left renal artery stent with a non-flow limiting focal area of stenosis midway through the stent. These findings were new in comparison to a CTA abdomen and pelvis obtained six months earlier prior to EVAR. An ultrasound Doppler of the abdomen and pelvis also showed the left renal artery stent with patent flow. Chest radiograph was unremarkable.

**CPC-EM Capsule**

What do we already know about this clinical entity?

*Secondary hyperaldosteronism presents with multiple metabolic abnormalities and can signify failure or occlusion of the post-abdominal aortic aneurysm repair stent*.

What makes this presentation of disease reportable?

*Secondary hyperaldosteronism secondary to renal artery thrombosis as well as a focally-stenosed renal artery stent following endovascular repair treatment for abdominal aortic aneurysm is novel*.

What is the major learning point?

*The metabolic derangements associated with hyperaldosteronism, metabolic alkalosis with hypokalemia, in those with renal artery stents must be recognized by the Emergency Medicine physician*.

How might this improve emergency medicine practice?

*Accurately diagnosing hyperaldosteronism and recognizing post-AAA repair complications of renal artery stenosis*.

The patient’s initial presentation was concerning for secondary hyperaldosteronism given his hypokalemia and metabolic alkalosis. Given the patient’s history of AAA status post EVAR and abnormal CTA results, vascular surgery was consulted. Similarly, endocrinology and nephrology were consulted due to metabolic derangement. In conjunction with the various consulting specialties, the patient was admitted for four days and was started on amlodipine, hydralazine, and spironolactone (which was up titrated during the hospital stay due to persistent hypertension and hypokalemia). At discharge, renin-aldosterone levels were pending but eventually resulted at 44.4 nanograms per milliliter per hour (ng/mL/hr) (reference range: supine = 0.2–1.6; upright = 0.5–4.0) for renin, and 56.0 nanograms per deciliter (ng/dL) (less than 16 ng/dL) for aldosterone. Upon discharge, all three medications above were continued, angiotensin-converting enzyme inhibitors and angiotensin II receptor blockers were recommended to be avoided, and the patient was scheduled for an outpatient captopril study.

## DISCUSSION

Aldosterone, a mineralocorticoid produced by the adrenal glands, exerts its effects throughout the body. However, the effects are most often targeted to the kidneys in the presence of low blood volume or electrolyte disturbances. The RAAS system is the principal regulator for the production and potentiation of aldosterone and is vital for survival.^5^ In short, the enzyme renin is secreted from the kidneys and acts on angiotensinogen (produced by the liver). The product is angiotensin I, which then is transformed into angiotensin II in the lungs. Angiotensin II can exert its own influence in the body through generalized vasoconstriction, increased proximal tubule reabsorption of sodium, stimulation of antidiuretic hormone secretion, and, most importantly, stimulation of aldosterone secretion.^4^ In the presence of decreased systemic arterial pressure, resulting in decreased glomerular filtration and renovascular pressure, or high serum potassium, aldosterone acts in the kidneys to reabsorb sodium, water, and excrete potassium.^4^

The case described above exhibits a clinical syndrome known as secondary hyperaldosteronism, in which the body produces excess aldosterone secondary to overactivation of the RAAS system.^6^ The excess aldosterone does not come from an aldosterone-producing tumor (which distinguishes primary from secondary) but rather from a high amount of renin secondary to causes such as renal artery stenosis, aortic coarctation, reninoma, pregnancy, or cirrhosis.^4^,^6^ Our patient had many potential sources of his secondary hyperaldosteronism given his newly diagnosed chronic right renal artery thrombosis and left renal artery stent with focal stenosis. While Doppler imaging showed adequate flow through his left renal artery, we postulate that his bilateral decreased renal perfusion contributed to over-activation of his RAAS system leading to a hyperaldosterone state.

Patients with excess aldosterone are prone to hypervolemia and hypertension as aldosterone influences the kidneys to reabsorb sodium and water to return volume levels to acceptable levels.^7^ Angiotensin II, from the activation of the RAAS system, can also contribute to hypertension and increased thirst, which were experienced by the patient. Hypokalemia and metabolic alkalosis are common in hyperaldosteronism and are a consequence of aldosterone’s action on the renal collecting tubules, leading to increased sodium reabsorption, which causes movement of cations (hydrogen and potassium) into the tubular lumen to maintain electrical neutrality.

Given the initial concern for the patient’s left renal artery stent stenosis contributing to his presentation, it is important to discuss other complications of AAA repair. Perioperative complications are similar between EVAR and OSR and include wound complications, renal failure, colonic ischemia, death, myocardial infarction, and pneumonia.^8^ Postoperative, long-term complications include endoleaks (leakage of blood between the graft and aneurysm sac; more common in EVAR than OSR), graft infection, aortoenteric fistula, buttock claudication and limb occlusion, and sexual dysfunction.^8^ Patients receiving EVAR are spared the ischemic insult of aortic cross-clamping and often have less perioperative hemorrhage, but one must consider the potential nephrotoxicity associated with intravenous contrast as well as manipulating the aorta in such a way that plaques can become disrupted and embolize into the renal vasculature.^9^ Unfortunately, renal failure is common post AAA repair and can have significant and long-lasting downstream effects on the body such as the metabolic derangement seen in our patient with secondary hyperaldosteronism.

The primary treatment of secondary hyperaldosteronism is with mineralocorticoid receptor blockade with spironolactone or eplerenone. Spironolactone is a nonselective mineralocorticoid receptor antagonist with binding ability to both androgen and progesterone receptors as well.^10^ The majority focus of spironolactone is the renal cortical collecting ducts as it acts as potassium sparing diuretic and antihypertensive drug.^11^ Spironolactone is dosed at 25–200mg/24hr through either oral suspension or tablet.^12^ Dosage can be titrated to goal blood pressure as needed outpatient. Side effects include gynecomastia, menstrual disturbances, and impotence due to its effects on androgen and progesterone receptors.^13^ In patients experiencing sexual side effects, eplerenone may be used in place.^14^

## CONCLUSION

As aldosterone has many effects on the human body, it is important for the emergency physician to consider the diagnosis of hyperaldosteronism in patients presenting with hypertension with hypokalemia and metabolic alkalosis. This is especially true in patients who have undergone EVAR of AAA and left renal artery stenting as this could signify failure or occlusion of the stent.
